# Analysis of spatiotemporal changes of agricultural land after the Second World War in Czechia

**DOI:** 10.1038/s41598-021-91946-1

**Published:** 2021-06-16

**Authors:** Vít Zelinka, Johana Zacharová, Jan Skaloš

**Affiliations:** 1 Faculty of Environmental Sciences, Czech University of Life Sciences Prague, Kamycka 129, 165 00 Prague, Suchdol, Czech Republic; 2 Faculty of Environment, Jan Evangelista Purkyně University in Ústí nad Labem, Pasteurova 3632/15, 400 96 Usti nad Labem, Czech Republic

**Keywords:** Ecology, Environmental impact

## Abstract

The term Sudetenland refers to large regions of the former Czechoslovakia that had been dominated by Germans. German population was expelled directly after the Second World War, between 1945 and 1947. Almost three million people left large areas in less than two years. This population change led to a break in the relationship between the people and the landscape. The aim of the study is to compare the trajectories of these changes in agricultural landscapes in lower and higher altitudes, both in depopulated areas and areas with preserved populations. This study included ten sites in the region of Northern Bohemia in Czechia (18,000 ha in total). Five of these sites represent depopulated areas, and the other five areas where populations remained preserved. Changes in the landscape were assessed through a bi-temporal analysis of land use change by using aerial photograph data from time hoirzons of 2018 and 1953. Land use changes from the 1950s to the present are corroborated in the studied depopulated and preserved areas mainly by the trajectory of agricultural land to forest. The results prove that both population displacement and landscape type are important factors that affect landscape changes, especially in agricultural landscapes.

## Introduction

The abandonment or change in the use of less fertile farmlands can be observed throughout Europe. Significant amounts of agricultural land (mainly arable land) have ceased being used for their non-original purposes at higher altitudes since the 1950s^1^. An interest in this issue has grown recently^2–4^. Also, thanks to the availability of aerial photographs and geographic information system (GIS) capabilities, many studies are focusing on landscape change and its drivers^5–11^. The depopulation of unhostile mountain regions is a frequent cause of landscape transformation, as the natural conditions such as especially climate and soil quality limit land use. Population growth in urban regions and population decline in rural regions as a result of net migration or natural population change can be observed across Europe^12^. Effect of the depopulation during the twentieth century on landscape structure have been studie in Mediterranean regions, such as in Spain^13^ and Italy^14^. The polarisation of the land uses between either intensification or abandonment directly in recent decades^15^, urban sprawl followed by growth of infrastructures and accompanied by losses of visual values of rural areas^16^ were frequently cited as reasons for landscape changes. And also in the Eastern European case, the so-called socialist industrialisation of agriculture in the post-war period^17,18^. Also, periods of social and economic unrest cause a decrease in the anthropogenic pressure on the landscape. Agricultural land abandonment can be described as the cessation of agricultural activities on arable land, meadows, and pastures with subsequent natural vegetation recovery^19^. This is characterised by a temporary and, in some cases, permanent turning in the development of the secondary landscape structure. That results in succession—scrub encroachment and forest regrowth on agricultural land^20,21^.

Historically, European rural landscapes have been affected by the after-effects of the Second World War, namely the expulsion of German communities from Central and Eastern Europe. There are several studies focused on depopulated areas in Poland, such as the Polish Carpathians^22,23^ or the Polish Sudetes mountains^24^. However, Germans were displaced after 1945 not only from Poland but also from Czechoslovakia, Hungary, Yugoslavia, Romania, and the Baltic countries^25^.

In Czechia, the trend of land abandonment is particularly relevant in the areas from which the German population was expelled directly after the Second World War, between 1945 and 1947. These population fluxes were concentrated in regions that had been dominated by German speaking popullation, called the Sudetenland. The term Sudetenland was originally used for one of the four territories spontaneously proclaimed by the German inhabitants of the border areas of Czechoslovakia after the collapse of the Habsburg Monarchy^26^. This term was later applied to large border areas of Czechoslovakia inhabited mainly by Germans. In the border region of the Sudetenland, a trend of decreasing anthropogenic land use intensity was observed in the study of Bičík and Kabrda^27^. Vast border regions became suddenly uninhabited, resulting in a significant decrease in arable land and the transition of land cover into forests, meadows, and pastures^17^.

The region affected by this historical process is a unique case for the following reasons. First, the demographic change came very quickly. Almost three million people left large areas in less than two years; this represented a quarter of the population of Czechoslovakia at the time. These vast regions suddenly became uninhabited. The territory of Czechoslovakia formerly occupied by Nazi Germany had an area of nearly 30,000 sq km^28^, an area comparable to the size of Belgium. Although there was a resettlement process, the population of these regions has never reached pre-war levels. Thus this process led to a decrease in agricultural land and an increase in forest areas^17^.

This demographic change affected all types of agricultural lands except the most fertile at the lowest altitudes. However, these areas cannot be described as marginal. This population change eliminated traditional methods of farming in various areas and led to a break in the relationship between the people and the landscape where they live^17,29^.

The spatial context of these demographic changes is also interesting. Due to the demarcated areas dominated by the German population^28^, there are often two settlements with different types of demographic developments in a single landscape type^30^: those which were almost completely depopulated, and on the other hand, those that were only marginally affected by this sudden drop in population.

Thus, this study focuses on the effects of post-war demographic changes on the development of agricultural lands. The aim is to compare the trajectories of these changes in agricultural landscapes, both in depopulated areas and areas with preserved populations, and to detect differences in lowland and highland landscapes. This could help to distinguish precisely the impact of sociological factors and natural conditions on agricultural land abandonment. Despite a slight slowdown in recent years^31^, similar trends of farmland abandonment continue to be widespread land-change processes in Europe. European regions with negative migration balances have higher probabilities of abandonment compared to regions with positive migration balances^32^.

Several studies are focusing on land-use change in depopulated areas^13,14,22,23^, but no studies are focusing on landscape changes and trajectories. Consequently, the main goal of this study is to fill this research gap by analysing landscape changes in depopulated areas and areas with preserved populations.

This research builds on the results of a study conducted in rural areas of higher altitudes in 2017^33^. The comparative study carried out in 2017 at higher altitudes of Czechia indicated large differences in the overgrowth of agricultural land by forests in the depopulated areas and in the areas that remained settled. Current paper adds information regarding the change trajectories of depopulated areas in more fertile lands at lower altitudes (landscapes of lowlands and highlands). The results of the study proved, that population displacement and landscape type are important factors affecting changes of agricultural landscapes. We conclude results of the study to show knowledge gap as the point to focus on within this manuscript.

The answers to questions below can serve as one of the bases in creating the concept of nature protection, landscape planning and subsidy policy in agriculture in the future.

The following research questions to be answered are:

- *What changes and trajectories of agricultural land exist in lowlands and highlands?* Answering this question will help us to determine the effect of the post-war population displacement on the landscape change in lowlands and highlands. Many mountain areas across Europe were depopulated in the second half of the twentieth century. In case of the Sudetenland, this depopulation was massive and fast, thus differences are expected and have not been described in detail yet in Czechia.
- *How do the trajectories of agricultural land affected by population expulsion differ from the trajectories of those which remained inhabited?* Our study focuses on agricultural land, which is a landscape component that is directly dependent on human management. A long-term change in population is always reflected in agricultural land changes. In extreme cases, there is a reversal of large-scale forest vegetation development. On the other hand, extensively farmed mosaic landscapes of a high biological value can develop. Therefore, the question above should also be answered, to reveal variety of change
- *What is the effect of depopulation on the landscape microstructure?* Changes of spatial configuration of agricultural land patches have not yet been described although shape and area of patches can play substatial role for landscape functioning. Thus, quantification of these changes can bring useful insights for landscape management.

## Data and methods

### Study area

#### Selection of study sites

**Figure 1:**
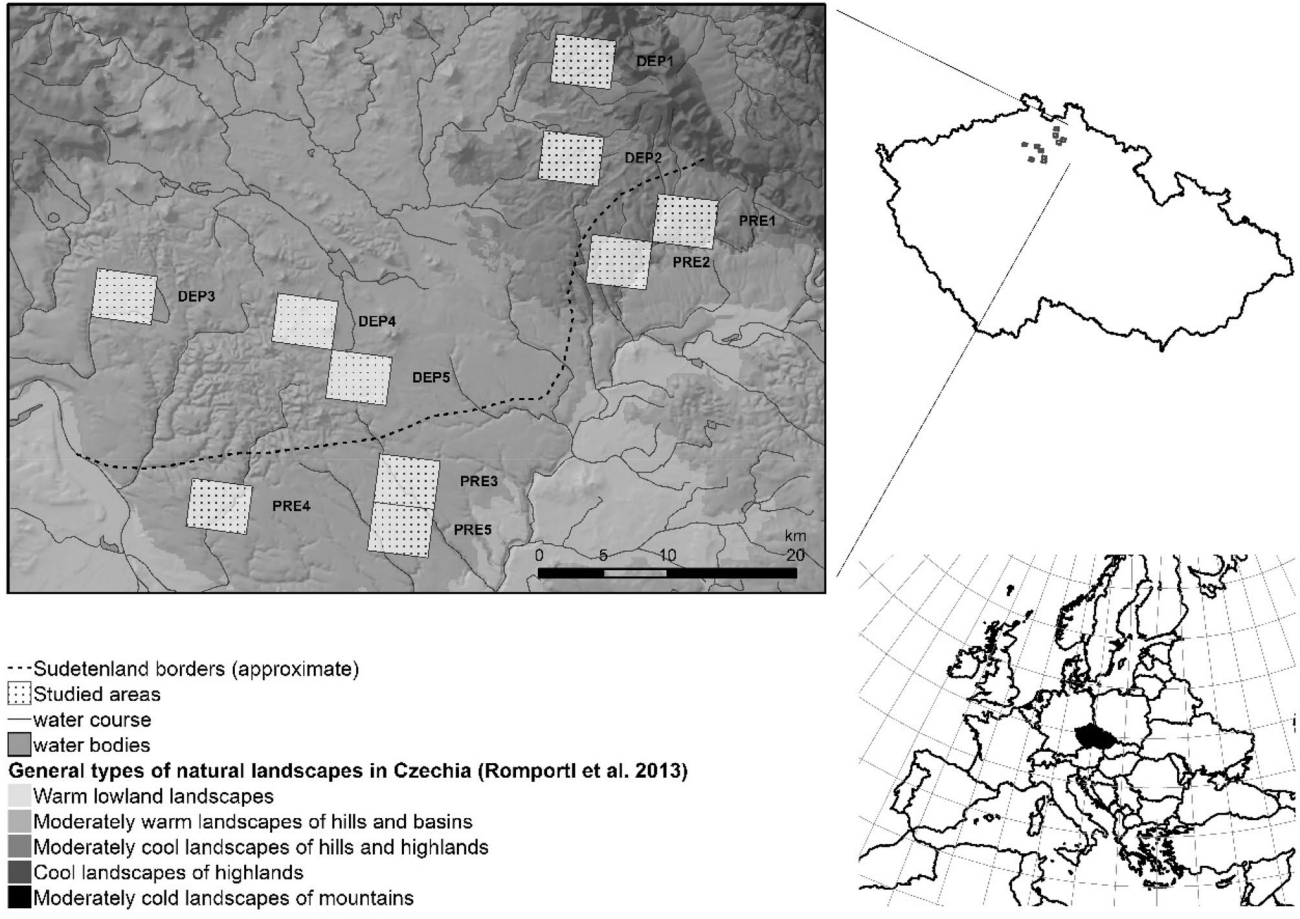
Location of the study sites in Northern Bohemia in Czechia. Study sites in the depopulated region are labelled DEP1–DEP5, sites in the region with preserved population are labelled PRE1–PRE5 (map created in the ArcGIS 10.6 software^34^).

This study included ten sites in the region of Northern Bohemia in Czechia (Fig. 1). These sites serve as good examples of areas that were depopulated after the Second World War as well as areas that were only marginally affected by this sudden drop in population. The study sites are located in the area where the boundaries between depopulated and undepolupatedregion form an elevation gradient. Therefore it is possible to compare areas with different demographic developments at different altitudes. This setting is relatively rare in Czechia.

#### Study site areas

Each of the ten rectangular study sites cover 1800 ha. Five of them represent depopulated areas, and the other five sites area where populations remained preserved.

#### Demographic characteristics

As a demographic dataset for this study, we used census data from 1 December 1930^35^. Based on the census demographic information, territories, where more than 80% of the population was made up by individuals of German nationality, were selected as depopulated areas. The areas with more than 80% of the Czechoslovak nationality population were chosen as preserved ones.

#### Landscape types

Landscape typology combines climatic and geomorphologic characteristics, such as average annual temperature, slope, and elevation; based on these factors, six general types of natural landscapes (hereinafter ‘landscapes types’) were defined by Romportl et al.^30^. Five study sites were chosen in each demographic area type (depopulated or preserved). Within each demographic grouping, three of the study sites were within the landscape type of *moderately warm landscapes of hills and basins* (hereinafter simply ‘lowlands’) and two in *moderately cool landscapes of hills and highlands* (hereinafter ‘highlands’) defined by Romportl et al.^30^. At least 80% of the area of each studied site belongs to the respective landscape type. The landscape types covered in this study are described in more detail below:

1. *Lowlands* Within this type of a natural landscape we studied six sites covering a total area of 10,800 ha (see Fig. 1)—three depopulated sites (a total of 5400 ha; study sites DEP3–DEP5 in Fig. 1) and three preserved sites (PRE3–PRE5 in Fig. 1).
2. *Highlands* We studied four highland sites with a total area of 7200 ha—two were depopulated sites (a total of 3600 ha; DEP1 and DEP2 in Fig. 1), and two were preserved sites (PRE1 and PRE2 in Fig. 1).

All of the studied sites are situated near the demarcated borderline of the area dominated by the German population and the area dominated by the Czechoslovak population before the Second World War^28^.

### Data sources

The change trajectories were analysed using GIS to compare agricultural land cover in 1953, representing the state of the landscape shortly after the displacement of the Sudeten Germans, with the current 2018 time horizon. Historical and contemporary land cover information was provided by aerial photographic images from 1953 and aerial orthophotos from 2016 to 2017. Additional field mapping was also carried out on selected sites in summer 2018.

#### Post-war time horizon (1953) and present time horizon (2018)

For the purposes of this study, the post-war time horizon is represented by black and white aerial orthorectified images from 1953^36^. The period defined by these two aerial photographs (1953 and 2018) represents a very dynamic period in the development of the landscape throughout Europe. By 1953, the movement of the German population had ended in the Sudetenland^28^. These black and white images reflect the landscape structure condition before the subsequent collectivisation of agriculture during the socialist régime in Czechoslovakia, which had its own large impacts at the landscape level^37,38^ and also at the level of single habitats sensitive to specific management^39^. During this period, double-tracked or multi-tracked development of the landscape also started. It means that some more distant and less fertile areas were abandoned, while agriculture intensified in more fertile areas^18^. This phenomenon can be observed from the second half of the twentieth century throughout Europe. In the conditions of the Czechia, later transition from a socialist planning economy to a market economy has worsened the process. In the displaced areas, however, the black and white images make it impossible to distinguish farmland from grasslands and arable land, because many fields have been transformed into a wasteland due to a lack of population. Nonetheless, these photos still generally capture the structure of the landscape during the pre-war period. For the current land cover determination (2018), we used aerial full colour orthorectified pictures from 2016 to 2017^40^. We carried out additional field mapping in August 2018.

### Data processing and analysis

Based on the aerial photograph data from 2016 to 2017 and the 1950s, vectorisation of polygons of selected land cover types was performed in the ArcGIS 10.6 software^34^. For vectorisation over those raster data, we used backward interpretation^41^. By using this method, overlay analyses do not produce sliver polygons, which are not real changes in the landscape^41^, and require further repairs and processing^42^. For purposes of this study, a special land cover category key was used. We distinguished ten types of land cover (see Table 1). As this study focuses on agricultural land, vectorisation was not performed in the entire studied territory, but only in areas with agricultural land present in 2018 or 1953. Similar approaches were taken in other studies such as Forejt et al.^39^, Demková and Lipský^38^, and Zelinka^33^. A spatiotemporal analysis of changes was performed in ArcGIS 10.6 using the overlay analysis tool Itersect^34^. The agricultural land patches were then sorted into three persistence categories, reflectingd their change trajectories and spatiotemporal changes, according to Skaloš et al.^43^.

#### Persistence categories

Changes in the landscape were assessed through an analysis of the land use change, which, using GIS tools, identifies different persistence categories of landscape segments of agricultural land. Detailed information on spatiotemporal changes in the landscape was then obtained by identifying the so-called change trajectories of the landscape patches. Following three categories are distinguished:

- *Continuous* (present both in 1953 and 2018).
- *Extinct* (present in 1953 but transformed into a different category of land cover by 2018).
- *Recent* (created by 2018 from a different category of land cover).

For vector data of both time horizons, the area of each polygon and land cover category was calculated using ArcGIS 10.6^34^.

### Monitored land cover categories

See Table 1.

**Table 1:** Land cover classification categories with codes used in change trajectories visualization.

Land cover category	Comments
Agricultural lands (AL)	The category aggregates arable land with meadows and pastures
Non-forest woody vegetation (NF)	Non-forest woody species vegetation in form of patches according to Demková and Lipský^38^. Linear elements (for example, alleys, riparian vegetation, vegetation along roads and railways, on balks etc.) are not taken into account
Forest areas (FOR)	Forest areas of a various age and origin
Water areas (WAT)	Water course and water bodies
Orchards, gardens, urban green (OG)	Intensively or extensively cultivated orchards and gardens. Also coniferous tree nurseries
Succession mixed cover (SUC)	Succession mixed cover of shrubs and herbaceous vegetation according to Raška et al.^44^
Rural roads (RR)	Unpaved rural and forest roads
Roads and railroads (ROA)	Paved roads, railways and paved parking areas
Built-up areas (BUI)	Residential and non-residential built-up areas, farmsteads, technical equipment warehouses and factories
Other areas (OAR)	Sports and industrial areas, agricultural facilities, cemeteries, landfills, quarries and dumps

### Data computation and statistical analysis

#### Analysis of the change and developmental of change trajectories

A model based on the Poisson distribution and a contingency table was used to assess differences of agricultural land patch numbers (various types of persistence), with respect to their location within two different landscape types (lowlands—LOW/highlands—HIGH) and regions of different demographic history (depopulated—DEP/preserved population—PRE). The effects of demography and landscape type were assessed by the share of particular persistence categories in study sites (after arcsin transformation of area A and shape index Si). To assess the overall level of dynamics related to the study sites, an index of agricultural land (AL) change was calculated as the ratio of the share of extinct AL to total AL.

#### Concerning landscape microstructure analysis

Linear models and analysis of variance (ANOVA) were used to assess the variability of the area (A) and the shape complexity of the patches (SI = shape index). Both of these values were derived from vector datasets (SI = Perimeter/√ π*Area). The values of the area and shape index were log-transformed for analysis. Patch numbers of this AL trajectory in the context of different landscape type and demography history were assessed by chi-square tests using a contingency table. Area and shape complexity was assessed in detail for agricultural land regrown by forest (trajectory paths of AL_FOR) using an ANOVA.

## Results

### Overall change

Change in the use of agricultural land in the studied sites between 1953 and 2018 was relatively high both in depopulated areas and in areas with preserved populations. In depopulated areas (DEP1–DEP5), there was a decrease in agricultural land from 5395.8 to 4651.3 ha during this period. This represents a decrease of approximately 14%. The mean share of continuous agricultural land was 51.3% of depopulated study areas. Meanwhile in the preserved population study sites (PRE1–PRE5), agricultural land area also decreased by approximately 8.4% from 6017.5 ha in 1953 to 5511.5 ha in 2018. Of these, 5394.9 ha fall within *continuous* areas. The agricultural land persistence for sites studied in depopulated areas and areas with preserved population is shown in Table 3. The agricultural land use is regarded as spatio-temporally stable.

**Figure 2:**
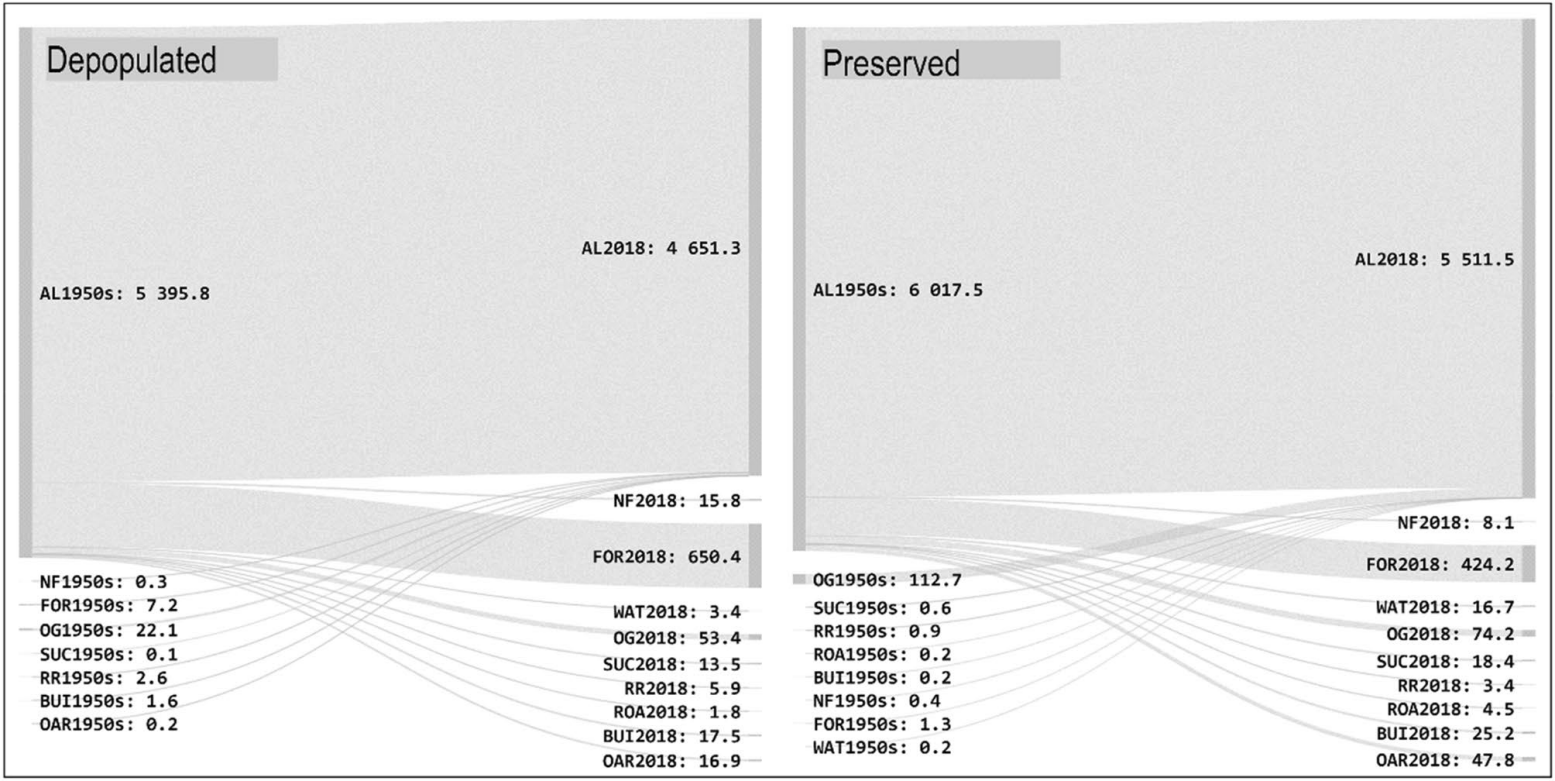
Overall change of agricultural land—change in trajectories between 1953 and the recent time horizon (in hectares). For land cover category codes see Table 1.

**Figure 3:**
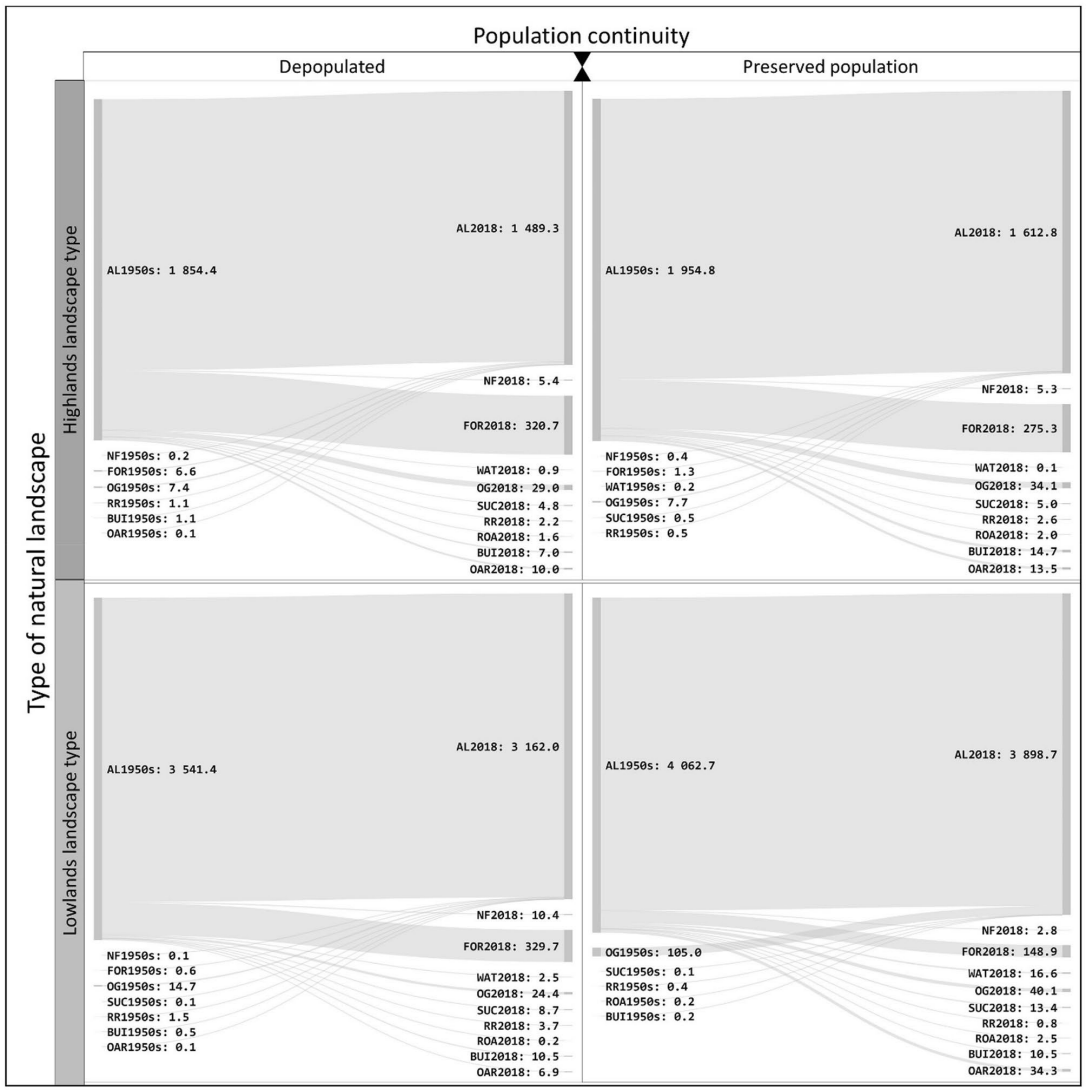
Agricultural land change trajectories between 1953 and the most recent time horizon in depopulated areas and in areas with preserved population considering their location in different landscape types (in hectares).

**Figure 4:**
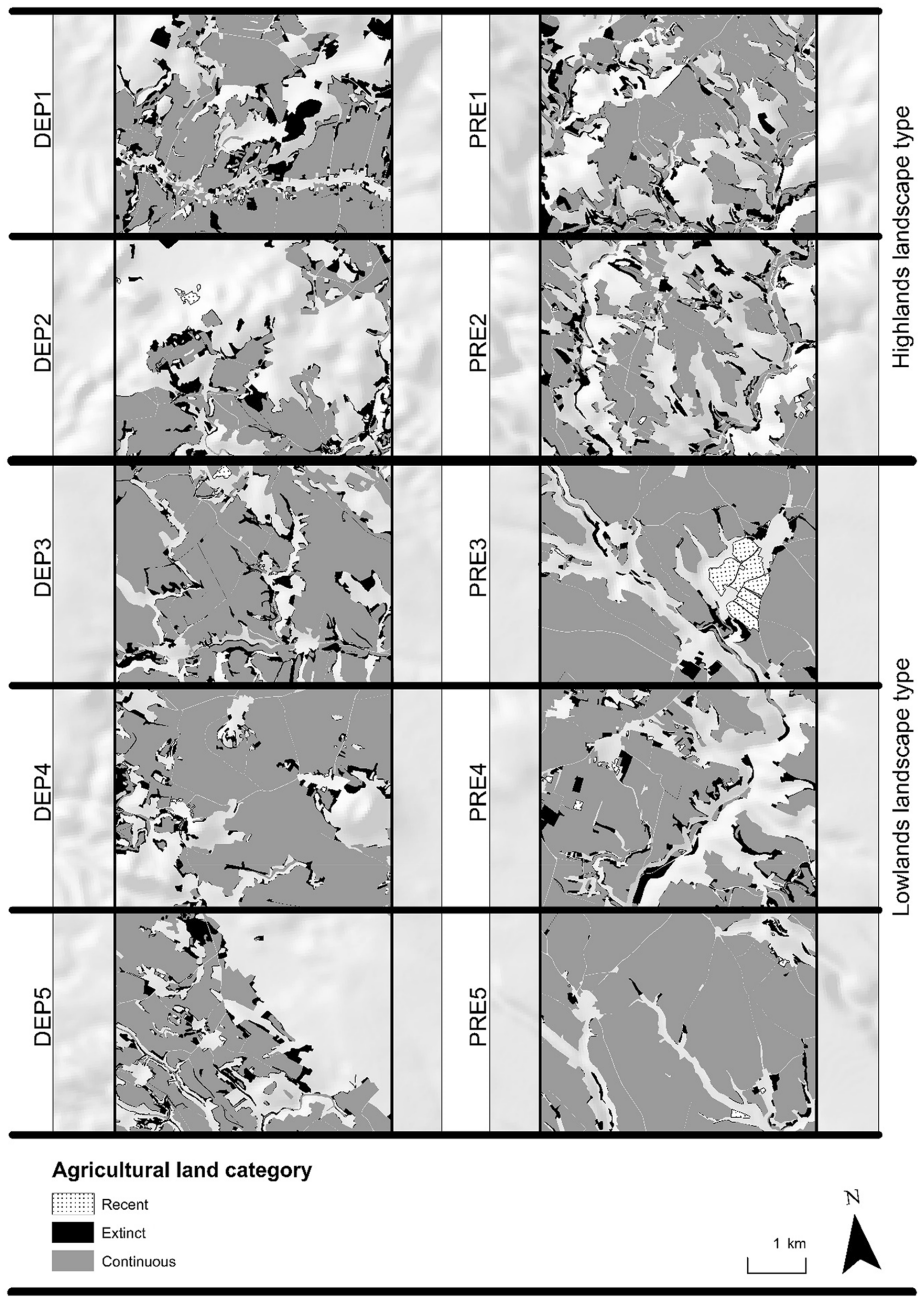
Spatial distribution of agricultural land persistence categories within the studied sites (maps created in the ArcGIS 10.6 software^34^).

As mentioned above, agricultural lands mapped in study sites in 2018 consist of both continuous (present in 1953 and 2018) and recent areas (created by 2018 in place of a different category of land cover). The share of *recent* agricultural land in the area of current (2018) agricultural land is 0.7% (34.1 ha) for depopulated areas and 2% (116.6 ha) in areas with preserved populations. The change trajectories of agricultural land in various persistence categories are shown in Sankey diagrams (Figs. 2 and 3). These diagrams show that the newly created agricultural land (recent) for both depopulated areas and areas with preserved population is predominantly made up of former orchards adjacent to settlements. The largest area of orchards converted into agricultural land between 1953 and 2018 is located in the Spikaly municipality within a region with preserved population, covering an area of 87.3 ha (study area PRE3; see Fig. 4). An exception is a relatively large area (5.4 ha) of agricultural reclamation of formerly forested areas in pits 6 and 7 of the Hamr II uranium mine, established in the 1980s (located in study area DEP2; see Fig. 4).

For depopulated areas, extinct patches of agricultural land make up a total of 778.6 ha and 622.5 ha for the areas with preserved population. The predominant type of change is the trajectory of *agricultural land—forest* (Fig. 2). This type of trajectory accounts for 84% of extinct agricultural land in depopulated areas and 68% in areas with preserved population.

### Effect of landscape type and demographic character on the persistence of agricultural land

**Figure 5:**
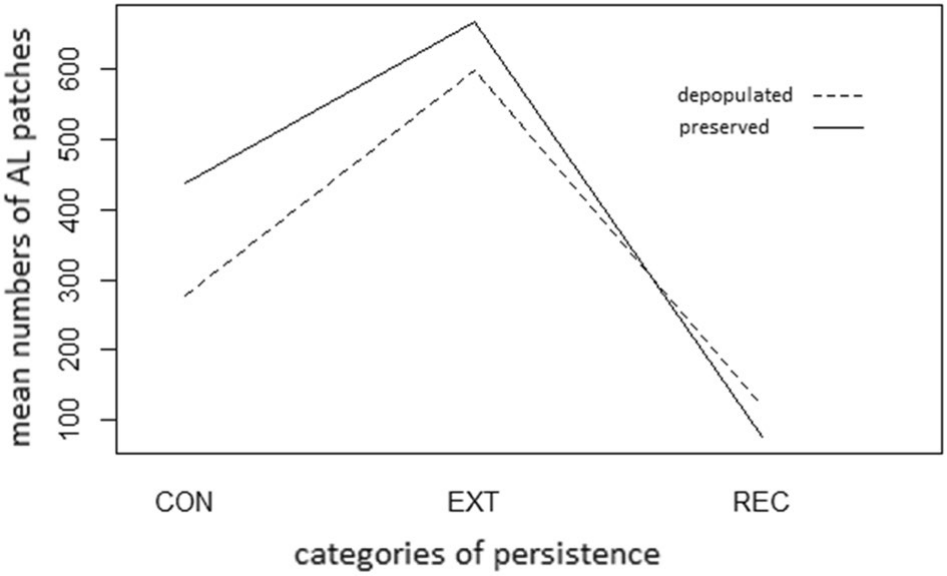
Mean numbers of AL patches in individual categories of persistence.

The results of the model glm (n ~ persistence category * demographic characteristics * landscape type, family = Poisson) suggest that the mean numbers of AL patches in categories of persistence (see Table 2 and Fig. 5) REC and EXT are significantly different from those in the continuous (CON) category (*p* = 0.01, *p* < 2*10^−16^, respectively). Similarly, the mean numbers of AL patches between the Sudetenland and regions with preserved population, and between the two considered landscape types differ as well (both *p* < 2*10^−16^).

**Table 2:** Mean numbers of AL patches in individual categories of persistence.

		DEP	PRE
**CON**			
LOW		382	384
HIGH		174	494
**EXT**			
LOW		635	566
HIGH		564	770
**REC**			
LOW		110	59
HIGH		129	95

Areas of particular persistence categories of agricultural land (continuous, extinct, and recent) in all of the study sites in depopulated areas and those with preserved population are shown in Table 3. For the spatial distribution of particular persistence categories within the study areas, see Fig. 4.

**Table 3:** Agricultural land persistence for depopulated areas (DEP1–DEP5) and areas with preserved population (PRE1–PRE5).

	ID	Agricultural land in 1953	Agricultural land in 2018		Agricultural land losses 1953–2018
		% Study area	Continuous	Recent	Extinct
			% Study area	% Study area	% Study area
Depopulated	DEP1	65.7	53.3	0.4	12.4
	DEP2	37.3	28.5	0.5	8.8
	DEP3	77.7	69.5	0.5	8.2
	DEP4	73.4	66.9	0.2	6.5
	DEP5	45.7	38.3	0.2	7.4
Preserved population	PRE1	60.0	49.7	0.2	10.3
	PRE2	48.6	39.4	0.4	9.3
	PRE3	73.6	69.1	5.0	4.5
	PRE4	66.6	58.4	0.6	8.2
	PRE5	85.5	83.2	0.3	2.2

#### The effects of demography and landscape types were assessed by the share of particular persistence categories in study sites (after the arcsin transformation of A)

The shares of patches of different persistence categories differ significantly among study sites for the continuous and extinct categories within landscape types (aov(arscin(%continuous) ~ demographic characteristics + landscape type: df = 1, F = 9.489, *p* = 0.0178; aov(arcsin(%extinct) ~ demographic characteristics + landscape type: df = 1, F = 19.424, *p* = 0.00313), but not by the category recent. According to the model results, different demographic histories do not affect the share of persistence categories within study sites (*p* < 0.05).

#### To assess the overall level of dynamics related to the study sites, an index of AL change was calculated as the ratio of extinct AL share to total AL share

**Figure 6:**
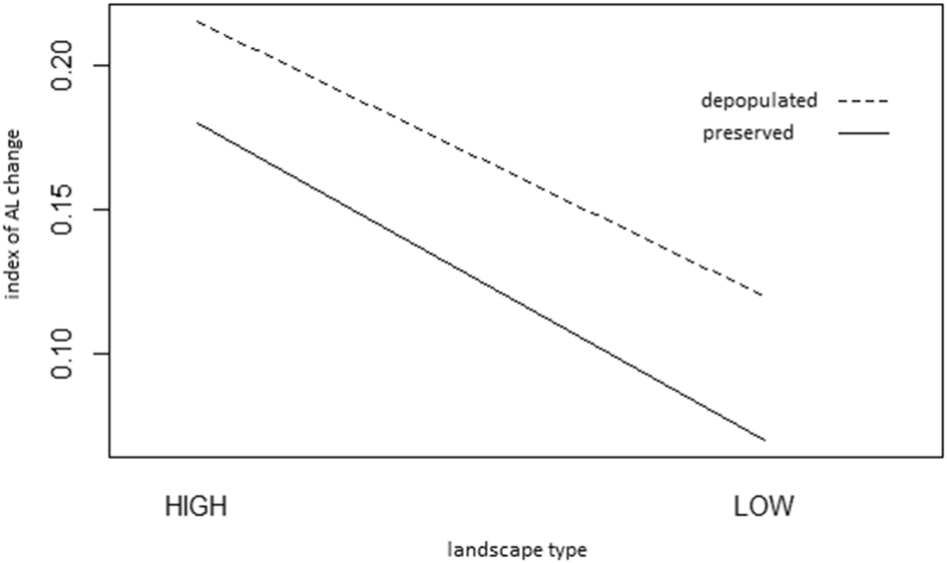
The level of dynamics in depopulated regions compared to regions with preserved population.

The level of dynamics in depopulated regions compared to regions with preserved population are higher based on Fig. 6. From Fig. 6 it is also evident that the mean index of change is higher in highlands and lower in lowlands.

### Landscape microstructure analysis

Based on the model, log(A) ~ persistence category + demographic characteristics + landscape type, it can be concluded that all the variables affect the aerial extent of AL patches (persistence category, demographic characteristics, landscape type; df = 2, F = 14.353, *p* = 6.13*10^−7^). The mean area of patches among persistence categories differ within landscape types (df = 2, F = 1253.5, *p* < 0.2*10^−16^), and also between former regions of the Sudetenland and Protektorát (df = 2, F = 7.914, *p* = 0.0004) (see Table 4).

**Table 4:** Mean area of patches among persistence categories.

Mean A in ha	CON	EXT	REC
DEP_HIGH	8.46	0.68	0.13
DEP_LOW	8.23	0.63	0.16
PRE_HIGH	3.24	0.46	0.11
PRE_LOW	9.90	0.48	1.80

Concerning the shape complexity with respect to the position of patches within regions of different demographic history in combination with two landscape types (aov(log(SI) ~ landscape type * demographic characteristics), we can conclude that the shape indices in the Sudetenland and outside of it differ significantly in lowlands (*p* = 0) but not in highlands (*p* = 0.82). We can also observe that the shape of AL varies significantly between landscape types in regions with preserved population (*p* = 10^−8^) but not within the former Sudetenland region (*p* = 0.07) (see Table 5).

**Table 5:** Shape complexity in respect to the position of patches within regions of different.

	DEP	PRE
HIGH	4.106532	4.014099
LOW	4.280266	3.646679

The results of the model log(SI) ~ persistence category * demographic characteristics * landscape type, focused on the assessment of patch shape complexity, can be summarised in the following way: the mean patch shape indices of AL differ significantly among persistence categories (df = 2, F = 190.635, *p* = 2*10^−16^) as well as among regions with different demographic histories (df = 1, F = 27.789; *p* = 1.42*10^−7^). At the same time, we conclude that the mean shape index of patches of different persistence categories differ significantly in context with landscape type (d = 2, F = 4.785, *p* = 0.00862) and also mean shape index of patches from depopulated regions and those with preserved population within the context of two landscape types (d = 1, F = 44.464, *p* = 2.91*10^−11^) (see Table 6).

**Table 6:** Mean shape index of patches of different persistence categories of AL.

MeanSI	CON	EXT	REC
DEP_HIGH	3.58	4.28	4.07
DEP_LOW	3.46	4.79	4.22
PRE_HIGH	3.31	4.43	4.33
PRE_LOW	3.07	4.06	3.44

### Overgrowth by woody vegetation as a typical way of agricultural land perishing in the Sudetenland

#### Patch numbers of this AL change trajectory within the context of different landscape types and demographic histories were assessed using the chi-square test and contingency table

**Figure 7:**
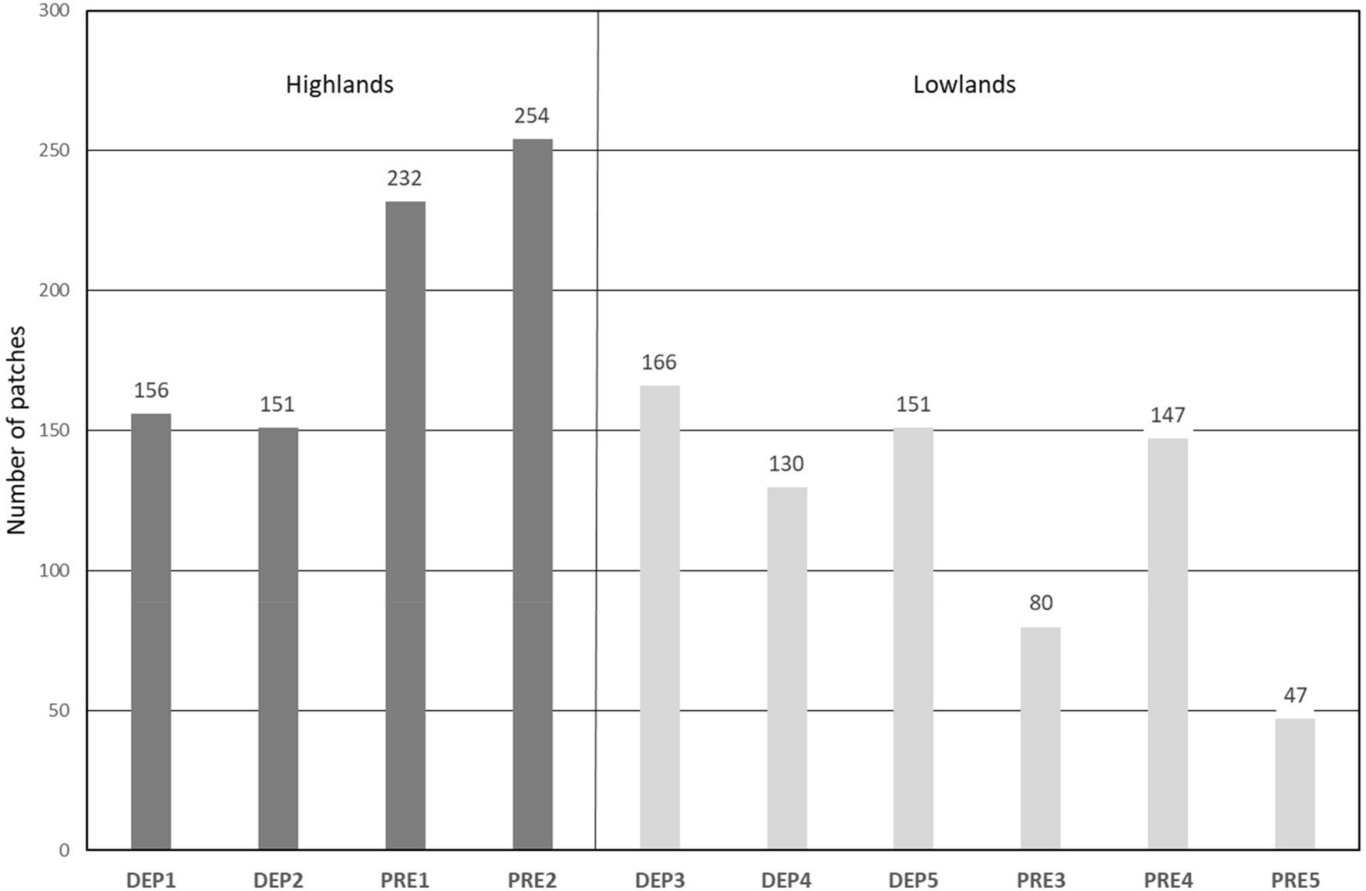
Number of patches with change trajectories of agricultural land—forest and agricultural land—non-forest woody vegetation in the depopulated and non-depopulated areas.

Test results show that both the landscape types and demographic characters of the regions affect the numbers of patches of this trajectory (*p* = 0.05), for details see Fig. 7.

#### Area and shape complexity was assessed in detail for agricultural land overgrown by forest (trajectory paths of AL_FOR) by using ANOVA

The results of models aov (log(A) demographic characteristics + landscape type) and aov (log(SI) ~ demographic characteristics * landscape type) show that the area and shape index of patches of AL regrown by forest are significantly affected by their location within or outside of the Sudetenland (area: df = 1, F = 5.972, *p* = 0.146; shape index: df = 1, F = 5.969, *p* = 0.147), but not by landscape type. For details see Table 7.

**Table 7:** Mean area and shape index of the patches with the trajectories of agricultural land—forest and agricultural land—non-forest woody vegetation in the depopulated and non-depopulated areas.

Study site	*DEP1*	*DEP2*	*DEP3*	*DEP4*	*DEP5*	*PRE1*	*PRE2*	*PRE3*	*PRE4*	*PRE5*
Mean A (ha)	1.14	0.94	0.70	0.79	0.73	0.59	0.55	0.40	0.62	0.55
Mean SI	4.44	4.38	4.76	4.60	5.04	4.61	4.37	5.12	4.34	4.32

**Figure 8:**
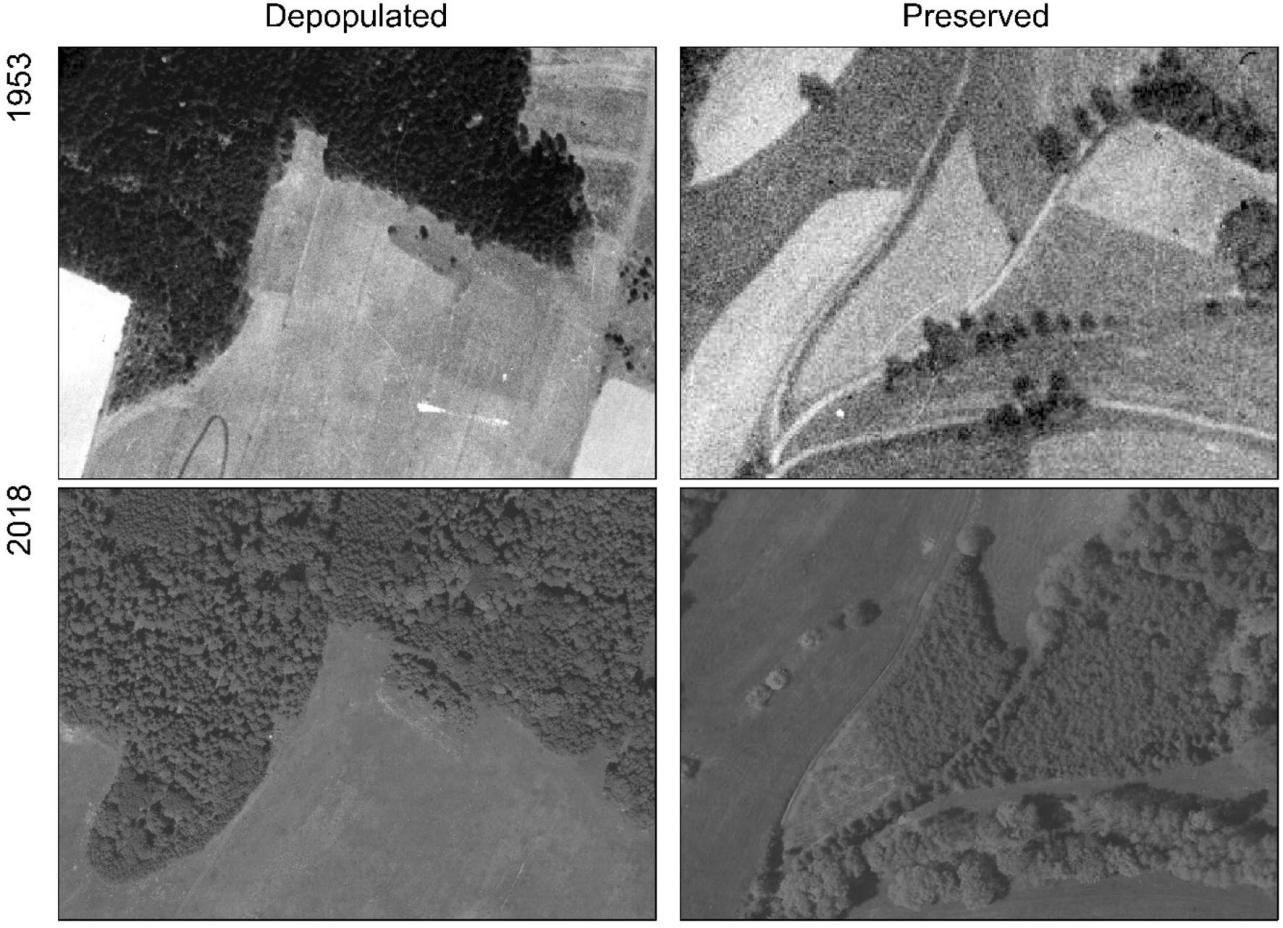
Noticeable visual difference in the dynamics of a new forest. Spontaneous succession and controlled afforestation (sources of aerial data: orthorectified images from 1953^36^, colour orthorectified images from 2016 to 2017^40^,maps generated in the ArcGIS 10.6 software^34^.

When looking at the areas of extinct agricultural land with the trajectory of *Agricultural land– forest* in depopulated and preserved areas, a certain visual difference is often obvious in the internal structure of the newly emerging forest. While the most common cause of this in depopulated areas is probably is the expansion of the forest to the detriment of earlier agricultural land by spontaneous succession, in preserved areas it is often controlled forestation (Fig. 8).

## Discussion

### Discussion on results

It is clear from the results of our analysis that declines in the area of agricultural lands occurred in depopulated areas as compared to the non-depopulated areas, particularly given to increases in the area of woody vegetation due to the extensification of agriculture as a result of population displacement^28^. Changes in land use from the 1950s to the present are thus corroborated in the studied depopulated and preserved areas mainly by the trajectory of *agricultural land–forest*. Similar studies showed this trend is discernible across Europe^13,22,32,45^ and corresponds with the known principles of cultural landscape dynamism^20,46^. The causes of abandonment of land differ. For the most part, long-term declines in population tied to migration to the urban areas and more fertile areas is perceptible^12^. However, this study concerns depopulated areas, where the gradual abandonment of the area is overshadowed by a rapid and mass decline in the population due to the forced displacement of the Germans after the Second World War^25,28^. When we compare the trajectory of extinct agricultural land in depopulated and preserved areas, the difference in the intensity of forest ingrowth in favour of depopulated areas is clear from the diagrams (Figs. 2 and 3). Similarly, this also applies to the comparison of the landscape types in a given area where the depopulated and preserved areas fall under the highlands landscape type, which manifest a substantially higher percentage of agricultural land transformed into forest land. The statistical processing performed here confirms that the average size of new forest patches is probably influenced by an area’s location in depopulated or preserved regions. In depopulated areas, the average size of the new forest patches to the detriment of agricultural land is larger as compared to non-depopulated areas, and the general sizes of new forest patches in depopulated areas are larger than in non-depopulated areas.

If we ignore these specific development trajectories and focus on the persistence of agricultural land, the difference between the depopulated and non-depopulated areas is less obvious. According to the linear model results, the size of agricultural land areas in various persistence categories exhibit a statistically significant difference between the lowlands and highlands landscape types. Also, location within depopulated or preserved areas does play a role according to the model results. Differences in the size of agricultural land areas between the lowlands and highlands landscape types may be because the character of the cultural landscape is a function of the environment. It is influenced by long-term geomorphological conditions in the form of the settlement of living organisms and disturbances^20^. The combination of these factors makes the character of settlement and mode of landscape management^47^, and thus also the shape of the agricultural areas, conditional. In this context, even the fact that the share of areas of persistence in the continuous and extinct categories in the total area of the model significantly differs between the landscape types is no surprise. Again, this can be easily explained by the different (generally smaller scale) agricultural use of the landscape in the highlands^48^. The shape index derived from vector datasets (SI = Perimeter/√ π*Area) of agricultural land regrown by forest (trajectory paths of AL_FOR) is influenced by the demography.

Based on comparisons of the quality of the models (AIC_DEMOGRAPHY_ = 3572.3 < AIC_LANDSCAPE TYPE_ = 3548.0), the shapes of landscape segments are to a large extent affected by their location in depopulated or in preserved areas rather than to the landscape type. This confirms the general principles of landscape ecology, where the main factor influencing the contrast in the landscape (and partly also the nature of the boundaries of landscape segments) is man (cultural landscape—high contrast, natural landscape—low contrast)^20^. For this reason, the influence of settlement is more significant in this case than the type of natural landscape. It is also obvious from the analysis that the extinct (EXT) and recent (REC) areas are, in terms of shape statistics, significantly different from the continuous areas (α = 0.05). This may be partly influenced by the results of the GIS analysis, which, to some extent, affect the shape of the landscape segments. On the other hand, this may be influenced by the different character and functioning of landscape segments given by the fact that they originated differently and have a different character in the landscape (continuous, recent).

### Discussions on methodology

It should be noted that the demographic divisions of the study territory do not exactly match the borders of the areas occupied by Nazi Germany in 1938. The main reason is that the German military occupation did not adhere to the boundaries of individual cadastral areas^49^. Thus, even part of the cadastral areas with a larger share of the Czech population also fell under the occupied territory. This created a string of small disputed areas along the border of the Sudetenland. However, our study sites are far enough away from this indefinite boundary.

Our study sites were chosen from a broader area, where the boundary between the depopulated and non-depopulated areas have transects across the belts of the landscape types proposed by Romportl et al.^30^. Thus, the landscape types used as the geographical framework of the study drew from both the lowlands and the highlands. This fact provides a unique opportunity to compare the responses of the landscape to the sudden depopulation in the transect from the most fertile areas of the lowlands to less fertile mountainous areas^50^. However, it is necessary to mention the fact that some of the study sites (DEP2 and DEP5) fall partially under the large stable forest areas (approximately 400 km^2^). Here, the typical matrix of the Czech agricultural landscape of the lowlands changes from a forest enclave in an agricultural landscape to enclaves of agricultural areas in the forest. These areas are characterised by poor soils, extremely sparse networks of watercourses, and other conditions^51,52^, and thus, are significantly different from the rest of the lowlands landscape type in which they fall. These are sparsely populated areas that correspond to colonisation failures in the Middle Ages^53^ and areas that were used long-term by the Soviet Army. In terms of accompanying facilities, infrastructure, and recultivation, these areas are also afflicted by the large-scale chemical mining of uranium ore from the 1960s to the 1980s. It is thus necessary to ask the questions of whether these two study sites can even be fully considered agricultural landscapes and to what extent these circumstances can influence the results of the analyses. As already stated in the results, DEP2 has a relatively large area of new agricultural land (5.4 ha) that results from the agricultural reclamation of formerly forested areas of pits 6 and 7 from the Hamr II uranium mine established in the 1980s. Information about this agricultural land, which became extinct in connection with the mining and military use of the area and is not related to the initial expulsion of the population, is difficult to corroborate. The use of aerial photographs from multiple periods and mapping of land use in the period before the post-war expulsion of population^54^ would, to a certain extent, contribute to the acquisition of such data.

The land cover categories used in this study were chosen with regard to the focus of the study^55^. This concerns the typical landscape cover of agricultural landscape types 2 through 5 as defined by Romportl et al.^30^. However, the use of this key seems to be problematic in some areas of the Sudetenland which, due to the local natural conditions, have a larger share of natural grasslands (e.g., dry grasslands) that freely build on the agricultural landscape. In these areas, it is not possible to expect the same response to depopulation as seen in the rest of the grasslands in the agricultural landscape because they are probably not primarily determined by human management^56^. In the conditions of the Czechia, these areas occur in the hottest and driest areas of the country, particularly in Northern, Central, and Eastern Bohemian as well as in South Moravia^57^. In the Sudetenland area, only the areas in the Louny Highlands are under consideration.

Depopulation or displacement of the population, is an important factor of landscape changes, which affects several landscape features and their characteristics. However, in the history of the landscape, as well as in history in general, we will be significantly influenced by the availability and nature of the source materials used: ‘we only see what the materials allow us’. Here, too, we observe these changes on the changes of landscape elements, which are well recognisable on the used substrates, i.e., woody plants. Unfortunately, it is more difficult to interpret permanent grassland, where the change would be much more noticeable. From the point of view of the model, collectivization processes in agriculture in the second half of the twentieth century can also be problematic. It is of course not possible to determine that the collectivization process took place in the same way in all municipalities. However in terms of land ownership, the development of the two areas can be considered similar. Mainly due to their proximity to each other. Since 1946, when controlled settlement took place, land has been allocated to new settlers. From 1948, however, the pressure from Moscow to implement uncompromising collectivization intensified significantly. This subsequently led to the implementation of collectivization into a law. These legal norms have been strictly applied in the whole territory of the then Czechoslovakia since 1950.

## Conclusions

A decrease in the area of agricultural land was recorded in both types of landscapes. However, the results of the study show that in the landscape where the population was displaced, there was an almost two-fold decrease in the area of agricultural land (from 14 to 8.4%). There is also a higher proportion of continuous habitats of agricultural land in relation to the size of the landscape parts, where there was no displacement of the population (60% versus 51.3%). A larger decrease in the area of agricultural land, a higher proportion of disappeared agricultural land enclaves, and a smaller proportion of continuous agricultural land habitats prove that population density and consequent displacement are significant factors of landscape changes that lead to extensification of land use. This is reflected in a reduction in the distribution of agricultural land and its greater spatiotemporal changes. If the areas of agricultural land disappear, it most often occurs in favour of forests and in the landscape affected by the displacement of the population (in 84% of the cases). This trend is also very common in a country unaffected by depopulation, but in a smaller number of cases (68%).

Landscape type has a statistically significant effect on the representation of continuous and extinct areas of agricultural land, but not on recent areas of agricultural land. On the other hand, the effect of displacement has not been proven. If changes are assessed by the index of changes, the magnitude of this change is higher in the landscape affected by displacement, and also in higher altitudes.

The results show that both factors (landscape type and the displacement of the population) significantly affect the number and average size of the areas. The average number of areas of new and extinct agricultural land categories is significantly different from the continuous agricultural land. The average number of patches of individual persistence categories of both displaced/not displaced and highlands/lowlands also differs statistically. Both the type of landscape and the displacement of the population statistically significantly affect the number of areas and their average size, i.e., they are strong factors that affect landscape management and thus the basic characteristics of landscape microstructure, which is influenced by landscape management.

In contrast to the landscape type, the shape of the patches is significantly affected by the displacement of the population in the lowland landscape. In a landscape unaffected by population displacement, the landscape type has a significant effect on the shape of the patches. The shape of the patches differs significantly in the individual development categories of patches of agricultural land, as well as between the landscape affected and unaffected by the displacement of the population. The shape of the patches also differs between individual landscape types and between landscapes affected and unaffected by population displacement.

The results prove that both population displacement and landscape type are important factors that affect landscape changes, especially agricultural landscapes. This is manifested on the one hand by a change in the area, the representation of individual developmental elements of agricultural land, and also by the basic characteristics of the landscape microstructure. Interestingly, we found that this effect is not as ‘strong’ and differs between the characteristics of the change within the landscape type.
